# Production, identification, *in silico* analysis, and cytoprotection on H_2_O_2_-induced HUVECs of novel angiotensin-I-converting enzyme inhibitory peptides from Skipjack tuna roes

**DOI:** 10.3389/fnut.2023.1197382

**Published:** 2023-07-12

**Authors:** Wang-Yu Zhu, Yu-Mei Wang, Ming-Xue Ge, Hua-Wei Wu, Shuo-Lei Zheng, Huai-Yu Zheng, Bin Wang

**Affiliations:** ^1^Cell and Molecular Biology Laboratory, Zhoushan Hospital, Zhejiang Province, Zhoushan, China; ^2^Zhejiang Provincial Engineering Technology Research Center of Marine Biomedical Products, School of Food and Pharmacy, Zhejiang Ocean University, Zhoushan, China; ^3^Ningbo Today Food Co., Ltd., Ningbo, China

**Keywords:** Skipjack tuna (*Katsuwonus pelamis*), roe, angiotensin-I-converting enzyme, peptide, endothelial cells, oxidative damage, cytoprotection

## Abstract

**Background:**

Exceeding 50% tuna catches are regarded as byproducts in the production of cans. Given the high amount of tuna byproducts and their environmental effects induced by disposal and elimination, the valorization of nutritional ingredients from these by-products receives increasing attention.

**Objective:**

This study was to identify the angiotensin-I-converting enzyme (ACE) inhibitory (ACEi) peptides from roe hydrolysate of Skipjack tuna (*Katsuwonus pelamis*) and evaluate their protection functions on H_2_O_2_-induced human umbilical vein endothelial cells (HUVECs).

**Methods:**

Protein hydrolysate of tuna roes with high ACEi activity was prepared using flavourzyme, and ACEi peptides were isolated from the roe hydrolysate using ultrafiltration and chromatography methods and identified by ESI/MS and Procise Protein/Peptide Sequencer for the N-terminal amino acid sequence. The activity and mechanism of action of isolated ACEi peptides were investigated through molecular docking and cellular experiments.

**Results:**

Four ACEi peptides were identified as WGESF (TRP3), IKSW (TRP6), YSHM (TRP9), and WSPGF (TRP12), respectively. The affinity of WGESF (TRP3), IKSW (TRP6), YSHM (TRP9), and WSPGF (TRP12) with ACE was −8.590, −9.703, −9.325, and −8.036 kcal/mol, respectively. The molecular docking experiment elucidated that the significant ACEi ability of WGESF (TRP3), IKSW (TRP6), YSHM (TRP9), and WSPGF (TRP12) was mostly owed to their tight bond with ACE’s active sites/pockets via hydrophobic interaction, electrostatic force and hydrogen bonding. Additionally, WGESF (TRP3), IKSW (TRP6), YSHM (TRP9), and WSPGF (TRP12) could dramatically elevate the Nitric Oxide (NO) production and bring down endothelin-1 (ET-1) secretion in HUVECs, but also abolish the opposite impact of norepinephrine (0.5 μM) on the production of NO and ET-1. Moreover, WGESF (TRP3), IKSW (TRP6), YSHM (TRP9), and WSPGF (TRP12) could lower the oxidative damage and apoptosis rate of H_2_O_2_-induced HUVECs, and the mechanism indicated that they could increase the content of NO and activity of superoxide dismutase (SOD) and glutathione peroxidase (GSH-Px) to decrease the generation of reactive oxygen species (ROS) and malondialdehyde (MDA).

**Conclusion:**

WGESF (TRP3), IKSW (TRP6), YSHM (TRP9), and WSPGF (TRP12) are beneficial ingredients for healthy products ameliorating hypertension and cardiovascular diseases.

## 1. Introduction

Marine creatures live in harsh marine habitats, causing them to have significantly different and more diverse proteins than those of terrestrial organisms (1, 2). The unique amino acid sequences hidden in marine proteins can be released by proteolytic hydrolysis and present a variety of biological activities, which provide multiple benefits to human health (2, 3). Presently, diverse bioactive peptides (BPs) have been produced from marine creatures and their processing byproducts, including bluefin leatherjacket (*Navodon septentrionalis*) (4, 5), Atlantic salmon (*Salmo salar*) (6), blue-spotted stingray (*Taeniura lymma*) (7), Skipjack tuna (*Katsuwonus pelamis*) (8), *Sardina pilchardus* (9), miiuy croaker (*Miichthys miiuy*) (10), monkfish (*Lophius litulon*) (11, 12), Siberian sturgeon (*Acipenser baerii*) (13), and shark (*Mustelus mustelus*) (14). Therefore, marine-derived BPs draw great attention to consumers and researchers due to their full possibility applied in functional foods and medicines (3, 15).

Hypertension is a clinic-familiar disease affecting the arteries of the human body, and uncontrolled hypertension becomes a huge potential risk of cardiovascular diseases (CVDs), atherosclerosis (AS), heart failure, stroke, and kidney diseases (16). In WHO’s report, about 1.28 billion people between 30 and 79 years worldwide have the disease, and the group number will increase to 1.56 billion in 2030 if it is not properly controlled. The causes of high blood pressure are intricate and multi-faceted among independent individuals, but evidences continue to confirm that family history, lack of exercise, tobacco use or vaping, excess alcohol consumption, certain chronic conditions, and obesity or overweight induced by a dietary imbalance were dominant factors to the increasing CVD populations (16, 17). Healthy lifestyle habits can help control hypertension, but severe patients must be treated with drugs (18). The oral drug is the common therapeutic measure to lower high blood pressure, and developing new drugs is the primary task to effectively control and manage hypertension population (19, 20). Angiotensin-converting enzyme (ACE) can inactivate the vasodilator bradykinin to up-regulate blood pressure via modifying angiotensin (Ang) I to active Ang II, then inhibition of ACE activity is a crucial approach to mediate systematic hypertension (21). Therefore, the synthetic ACE inhibitory (ACEi) drugs, including captopril (Cap), lisinopril and enalapril, have been used clinically to treat hypertension, endothelial dysfunction, and diabetic nephropathy, but these ACEi drugs show serious side effects and require careful prescription management (20, 22). Therefore, the search for safer ACEi drugs from natural resources can provide feasible alternatives to synthetic ACEi drugs for treating hypertension and CVDs.

Presently, some natural ACE inhibitors, including quinones, flavonoids, polyphenols, sesquiterpenoids, and alkaloids have been prepared from different creatures (23, 24), and ACEi peptides from animals, plants and microbes arouse the concern of consumers and researchers because their medical and nutritional benefits (25). Because of the particularity and diversity of marine proteins, ACEi peptides have been generated from different marine creatures, such as rainbow trout (*Oncorhynchus mykiss*) (26), *Mytilus edulis* (17), *Ruditapes philippinarum* (27), lizard fish (*Saurida elongata*) (28), jellyfish (*Rhopilema esculentum*) (29), Nile tilapia (*Oreochromis niloticus*) (30), *Okamejei kenojei* (31), *Salmo salar* (32), etc. Those ACEi peptides possess high potential to serve as anti-hypertension ingredients applied in diet or clinical therapy (21, 33).

Tuna and tuna-like species with catches of 8.0 × 10^6^ tons/year are one of the four most highly valuable catch groups worldwide, but their byproducts, composed of viscera, bones, heads and skins, occupy about 70% of processed fish in the factory process, which lead to serious financial losses and environmental contamination (34). Developing active ingredients or products using tuna byproducts is a delightful choice to reduce economic damage, protect the ecological environment, and provide quality products to consumers (34). Therefore, some BPs have been generated from different tuna and its byproducts, such as scale (35), muscle (36, 37), cardiac arterial bulbs (38–40), bone/frame (11, 41), skins (42, 43), milts (2, 44), and head and viscera (45, 46). In previous research, antioxidant peptides, such as YEA, ICRD, GEC, AEHNH, AEM, QDHKA, and YVM have been isolated from Skipjack tuna (*Katsuwonus pelamis*) roes and showed significant activity (47, 48). To make more efficient use of tuna roes, the objectives of this research were to produce and identify ACEi peptides from protein hydrolysate of Skipjack tuna roes. Moreover, the cytoprotection of prepared ACEi peptides on endothelial cells (ECs) against oxidative damage was systematically discussed.

## 2. Materials and method

### 2.1. Materials

Skipjack tuna roes were provided by Ningbo Today Food Co., Ltd. (Zhejiang, China). HUVECs were purchased from the Cell Bank of Type Culture Collection of the Chinese Academy of Sciences (Shanghai, China). ROS (Product no. E004-1-1), Hoechst33342 (Product no. G023-1-1), CAT (Product no. A007-1-1), SOD (Product no. A001-3-2), GSH-PX (Product no. A005-1-2), MDA (Product no. A003-1-2), ET-1 (Product no. H093-1-2), and NO (Product no. A013-2-1) kits were purchased from Nanjing Jiancheng Bioengineering Institute (Jiangsu, China). Glutathione (GSH), trypsin, pepsin, papain, ACE and FAPGG were purchased from Sigma-Aldrich (Shanghai) Trading Co., Ltd. (China). Alcalase, MTT, DMEM, DMSO, fetal bovine serum (FBS), norepinephrine (NE), trifluoro acetic acid (TFA), neutrase and Cap were purchased from Beijing Solarbio Science & Technology Co., Ltd. (China). ACEi peptides of WGESF (TRP3), IKSW (TRP6), YSHM (TRP9), and WSPGF (TRP12) with purity higher than 95% were synthesized in Shanghai Apeptide Co. Ltd. (China).

### 2.2. Determination of ACEi activity

The ACEi activity was measured by employing FAPGG as the substrate with the following modifications reported by Zhao et al. (18). In brief, the initial assay volume consisted of 50 μL of the substrate (3 mM), 50 μL of the ACE enzyme solution containing 1.25 mU of declared enzyme activity, and 50 μL of assay sample. All these solutions were incubated for 30 min at 37°C in a water bath first without mixing and then for an additional 30 min after mixing. ACE activity was stopped by 150 μL of glacial acetic acid. After that, the reaction mixture was separated by HPLC at 228 nm to determine the hippuric acid (HA) content produced due to ACE activity on the substrate. The control reaction mixture contained 50 μL of buffer instead of the assay sample and the control was expected to liberate the maximum amount of HA from the substrate due to uninhibited ACE activity. The percent inhibition of ACE activity was calculated as follows:


$$\mathrm{Inhibition activity}\;\left(\%\right)=\left[\left({\mathrm{HA}}_{\mathrm{control}}-{\mathrm{HA}}_{\mathrm{sample}}\right)/{\mathrm{HA}}_{\mathrm{control}}\right]\times 100\%.$$

The IC_50_ value is the concentration of peptide inhibiting 50% activity of ACE.

### 2.3. Preparation of roe hydrolysate of skipjack tuna

The preparation of tuna roe hydrolysate was performed according to the previous method (17). The degreasing process of Skipjack tuna roes was performed according to the described method by Wang et al. (48). The defatted tuna roes were separately hydrolyzed by alcalase (55°C, pH 8.5), neutrase (55°C, pH 7.0), flavourzyme (50°C, pH 7.0), papain (55°C, pH 7.0), pepsin (37.5°C, pH 2.0), and trypsin (37.5°C, pH 7.8) with enzyme dose of 2% (w/w) for 90, 120, 150, 180, 210, 240, or 270 min. After the hydrolysis reaction, the hydrolysates were heated in boiling water for 10 min to inactivate the proteases. Each hydrolysate was centrifuged at 10,000 g for 20 min, and the supernatants were freeze-dried and stored at −20°C. The tuna roe hydrolysate produced by flavourzyme showed the highest ACEi activity and was named STRH.

### 2.4. Separation of ACEi peptides from STRH

**Figure 1:**
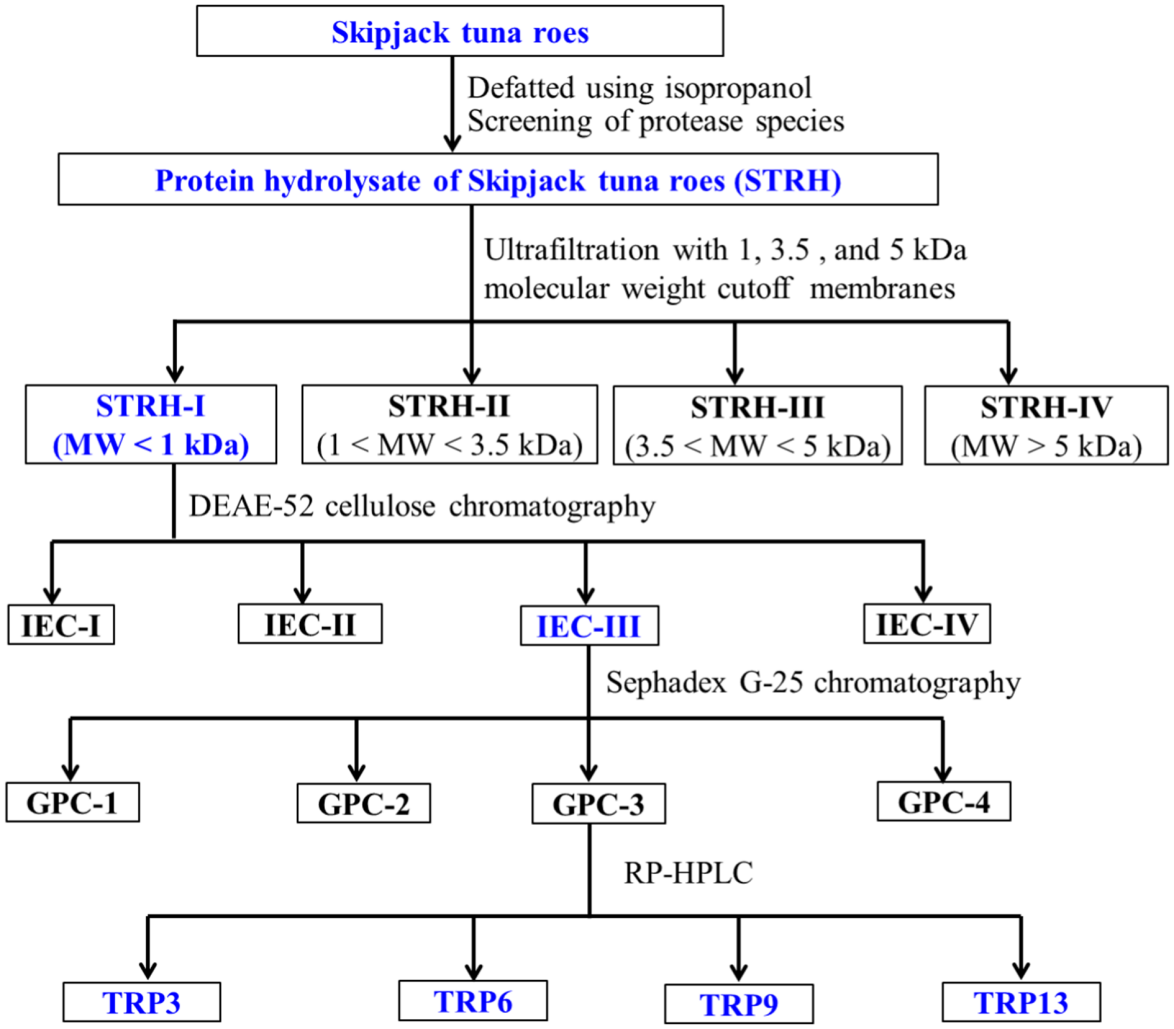
Flow diagram of purifying ACEi peptides from protein hydrolysate (STRH) of Skipjack tuna roes prepared using flavourzyme.

ACEi peptides were purified from TMPH using the following designed process (Figure 1).

#### 2.4.1. Ultrafiltration

STRH (100.0 mg/mL) was fractionated with 1.0, 3.5, and 5.0 kDa molecular weight (MW) cutoff membranes, and four fractions including STRH-I (<1 kDa), STRH-II (1–3.5 kDa), STRH-III (3.5–5 kDa), and STRH-IV (>5 kDa) were collected and freeze-dried. STRH-I had the maximum ACEi ability and was selected for the next experiment.

#### 2.4.2. Purification of ACEi peptides from STRH-I by chromatography methods

STRH-I (8.0 mL, 45.0 mg/mL) was added to the pre-treated DEAE-52 cellulose column (3.8 × 150 cm) and eluted in succession using 900 mL deionized water, 0.25 M, 0.50 M, and 1.0 M NaCl solutions at a flow rate of 3.0 mL/min. Each collected eluate (9.0 mL) was monitored at 220 nm and four fractions (IEC-I to IEC-IV) were collected and freeze-dried.

IEC-III (6.0 mL, 45.0 mg/mL) was purified by a Sephadex G-25 column (2.6 × 150 cm) and eluted with ultrapure water under a flow rate of 1.0 mL/min. Each collected eluate (3.0 mL) was monitored at 220 nm and four subfractions (GPC-1 to GPC-4) were isolated from IEC-III and lyophilized.

GPC-3 (25 μL, 80.0 μg/mL) was separated by RP-HPLC on a Zorbax 300SB-C18 column (9.4 × 250 mm, 5 μm) with a linear gradient of acetonitrile (10–50% acetonitrile in 30 min) inside 0.05% TFA at 1.2 mL/min. The eluate absorbance was monitored at 220 nm. Fifteen ACEi peptides (TRP1 to TRP15) were collected, lyophilized, and measured their activity.

### 2.5. Identification of ACEi peptide of TRP3, TRP6, TRP9, and TRP12

The sequences of TRP3, TRP6, TRP9, and TRP12 were analyzed by employing an Applied Biosystems 494 protein sequencer (Perkin Elmer, United States) (10). The MWs of TRP3, TRP6, TRP9, and TRP12 were detected by an ESI-Q-TOF-MS (Micromass, Waters, United States) (49).

### 2.6. Molecular docking experiments of TRP3, TRP6, TRP9, and TRP12

The crystal structures of captopril (Cap) and human ACE-lisinopril complex (1O8A.pdb) were gained from the RCSB PDB Protein Data Bank (PDB code: 1UZF). The Chimera software was used to confirm the position and size of the binding pocket by analyzing the interaction between ACE and peptide (TRP3, TRP6, TRP9, or TRP12). Non-standard residues in 1UZF model were deleted, and PDB files were converted into PDBQT files by the Autodock Tools. ACEi peptides (TRP3, TRP6, TRP9, and TRP12) were converted into SMILES format by the PepSMI tool, 3D models were drawn by the Discovery Studio program, and energy was minimized using steepest descent and conjugate gradient techniques. Molecular docking and free energy calculation were performed using the Autodock Vina. The best-ranked docking poses of TRP3, TRP6, TRP9, and TRP12 in ACE were captured on the binding-energy values and scores.

### 2.7. Effects of TRP3, TRP6, TRP9, and TRP12 on HUVECs

#### 2.7.1. HUVECs culture

HUVECs were cultured in DMEM supplemented with 100 g/mL streptomycin, 10% FBS (v/v), and 100 U/mL penicillin at 37°C in a humidified 5% CO_2_ atmosphere for 24 h.

#### 2.7.2. Cell viability, NO, and ET-1 determination

The viability of HUVECs treated by TRP3, TRP6, TRP9, and TRP12 was determined using MTT method. HUVECs were seeded in the 96-well plates, cultured for 24 h, and treated with ACEi peptides (100, 200, and 300 μM) at 37° C for 24 h. MTT (final content of 2 mg/mL) was added into cell culture. After 4 h, DMSO was added into each well and monitored at 490 nm. The cell viability (% control) was calculated.

After treating with ACEi peptides (TRP3, TRP6, TRP9, or TRP12, respectively) for 24 h, NO and ET-1 contents were separately determined by employing human NO and ET-1 assay kit as per manufactures’ protocol (18).

### 2.8. Cytoprotection of TRP3, TRP6, TRP9, and TRP12 on H_2_O_2_-damaged HUVECs

#### 2.8.1. Protection of TRP3, TRP6, TRP9, and TRP12 on H_2_O_2_-induced HUVECs

The oxidative damage model of HUVECs was established according to the previous method (50). HUVECs were seeded in the 96-well plates, cultured for 24 h, and treated with 300 mM H_2_O_2_ at 37°C.

The HUVECs were cultured for 24 h in a 96-well plate. Subsequently, the supernatant was aspirated and 20 μL of GSH (200 μM) and ACEi peptides (100 or 200 μM) were added in the protection groups, respectively. After 24 h, ACEi peptides were removed and H_2_O_2_ with the final concentration of 300 μM was added into the damage and protection groups for incubating 24 h.

#### 2.8.2. Measurement of levels of ROS, SOD, GSH-Px, NO, and MDA

The level of ROS was determined using DCFH2-DA assay and expressed as % control (50). The levels of SOD (U/mgprot), GSH-Px (U/mgprot), NO (μmol/L), and MDA (nmol/mgprot) were measured using assay kits according to manufacturer’ protocols.

#### 2.8.3. Morphological observation of HUVECs

Cell treatments with ACEi peptides, GSH, and H_2_O_2_ were according to the above method (50). The morphology of HUVECs was observed and photographed using an inverted microscope (Nikon Corporation, Kyoto, Japan). The percentage of apoptotic HUVECs was analyzed using previous methods (17, 50).

### 2.9. Data analysis

All data are expressed as the mean ± standard deviation (SD, *n* = 3) and analyzed by SPSS 19.0. An ANOVA test with Dunnett or Tukey test was employed to carry out the significant difference analysis (*p* < 0.05, *p* < 0.01, or *p* < 0.001).

## 3. Results

### 3.1. Preparation of protein hydrolysate of Skipjack tuna roes

**Figure 2:**
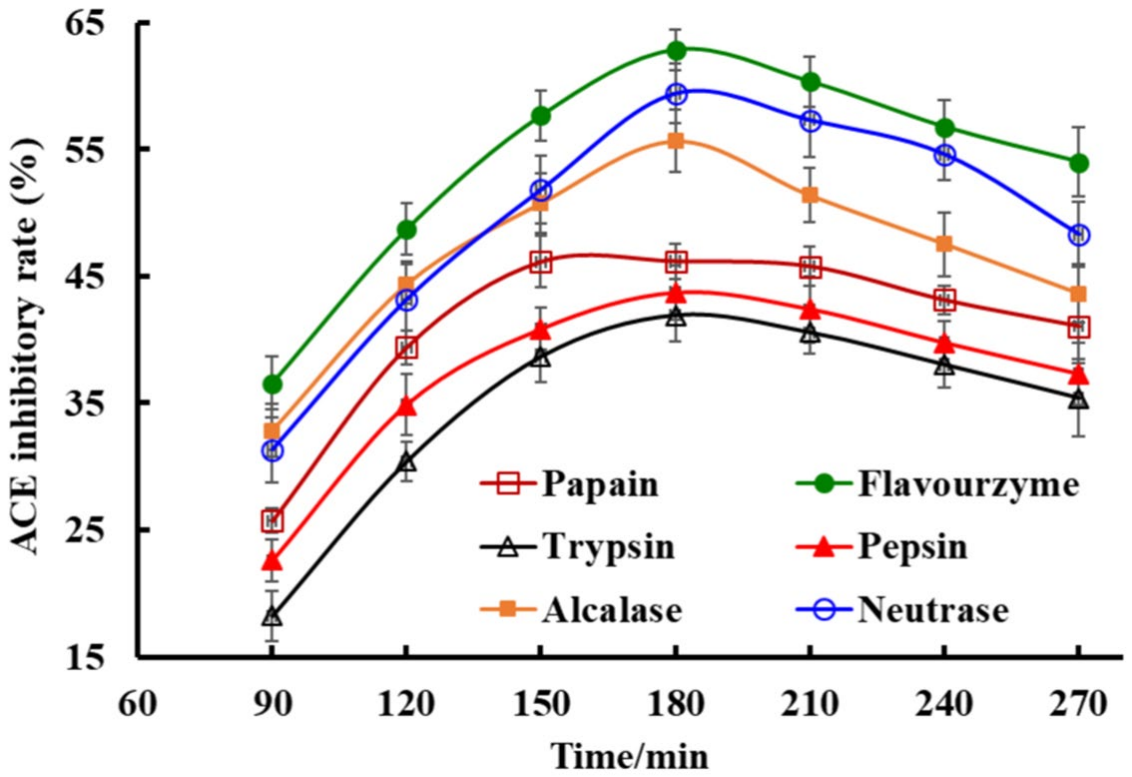
Effects of protease species and hydrolysis time on ACE inhibitory (ACEi) rates of Skipjack tuna roe hydrolysates at 3.0 mg/mL. All data are presented as the mean ± SD of triplicate results.

Proteins of Skipjack tuna roes were hydrolyzed by six proteases and the ACEi rates of generated hydrolysates were shown in Figure 2. The kind of proteases and hydrolysis time significantly influenced the ACEi rates of tuna roe hydrolysates. In addition, the ACEi rates increased gradually when the hydrolysis time ranged from 60 min to 180 min, but subsequently decreased after 180 min. Moreover, the tuna roe hydrolysate with the maximum ACEi rate (62.87 ± 1.98%) was produced by flavourzyme for 180 min, and the hydrolysate (STRH) was selected for the preparation of ACEi peptides.

### 3.2. Preparation of ACEi peptides from STRH

#### 3.2.1. Ultrafiltration

Through 1.0, 3.5, and 5.0 kDa ultrafiltration membranes, STRH was fractionated into four different MW peptide fractions, including STRH-I (<1 kDa), STRH-II (1–3.5 kDa), STRH-III (3.5–5 kDa), and STRH-IV (>5 kDa). At 1.5 mg/mL, the ACEi activity of STRH-I was 46.27 ± 2.49%, which was significantly (*p* < 0.05) higher than those of STRH (37.83 ± 2.07%), STRH-II (36.49 ± 1.85%), STRH-III (30.79 ± 1.28%), and STRH-IV (23.14 ± 0.97%), respectively. Then, STRH-I with the smallest MW was further isolated by chromatographic methods.

#### 3.2.2. Chromatographic separation of STRH-I

**Figure 3:**
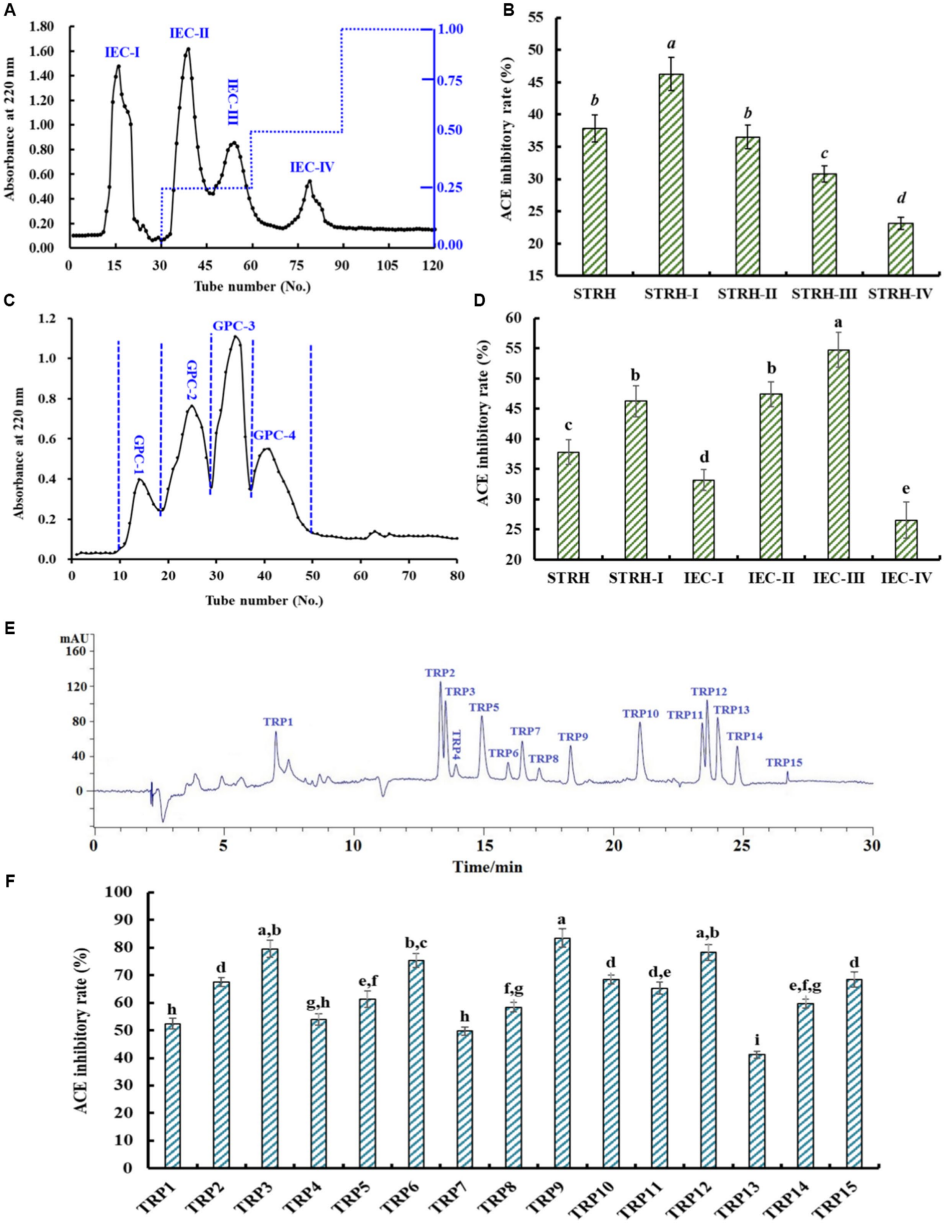
Chromatographic separation of STRH-I and ACEi rates of isolated fractions at 1.5 mg/mL. **(A)** Chromatogram profile of STRH-I isolated by DEAE-52 cellulose column, **(B)** ACEi rates of fractions (IEC-I to IEC-IV) from STRH-I, **(C)** Chromatogram profile of IEC-III isolated by Sephadex G-25 column, **(D)** ACEi rates of fractions (GPC-1 to GPC-4) from IEC-III, **(E)** HPLC profile of GPC-3 at 220 nm, and **(F)** ACEi rates of peptides (TRP1-TRP15) from GPC-3. ^a–i^Values with same letters indicated no significant difference (*p* > 0.05). All data are presented as the mean ± SD of triplicate results.

Using a DEAE-52 cellulose column, four fractions (IEC-I, IEC-II, IEC-III, and IEC-IV) were isolated from STRH-I (Figure 3A) and their ACEi rates were depicted in Figure 3B. At 1.5 mg/mL, the ACEi rate of IEC-III was 54.76 ± 2.87%, which was significantly (*p* < 0.05) higher than those of IEC-I (33.19 ± 1.76%), IEC-II (47.44 ± 2.05%), and IEC-IV (26.57 ± 3.02%), respectively (Figure 3B).

Using the Sephadex G-25 column, four peptide fractions (GPC-1, GPC-2, GPC-3, and GPC-4) were prepared from IEC-III (Figure 3C) and their ACEi rates were displayed in Figure 3D. At 1.5 mg/mL, the ACEi rate of GPC-3 was 63.75 ± 3.06%, which was significantly (*p* < 0.05) higher than those of GPC-1 (36.02 ± 1.94%), GPC-2 (50.23 ± 2.78%), and GPC-4 (27.49 ± 1.25%), respectively (Figure 3D).

Finally, GPC-3 was purified by RP-HPLC (Figure 3E). According to the chromatogram of GPC-3 at 220 nm, fifteen major components (TRP1–TRP15) with retention time of 6.97 (TRP1), 13.38 (TRP2), 13.53 (TRP3), 13.91 (TRP4), 14.95 (TRP5), 15.96 (TRP6), 16.46 (TRP7), 17.18 (TRP8), 18.29 (TRP9), 21.07 (TRP10), 23.41 (TRP11), 23.65 (TRP12), 24.06 (TRP13), 24.89 (TRP14), and 26.85 min (TRP15) were collected and freeze-dried. At 1.5 mg/mL, the ACEi rates of TRP3, TRP6, TRP9, and TRP12 were 69.54 ± 3.09%, 75.41 ± 2.56%, 83.49 ± 3.42%, and 78.24 ± 2.81%, respectively, which were significantly higher than those of other eleven isolated ACEi peptides (*p* < 0.05) (Figure 3F). Consequently, TRP3, TRP6, TRP9, and TRP12 were chosen for sequence identification.

### 3.3. Sequences and MWs determination of TRP3, TRP6, TRP9, and TRP12

**Figure 4:**
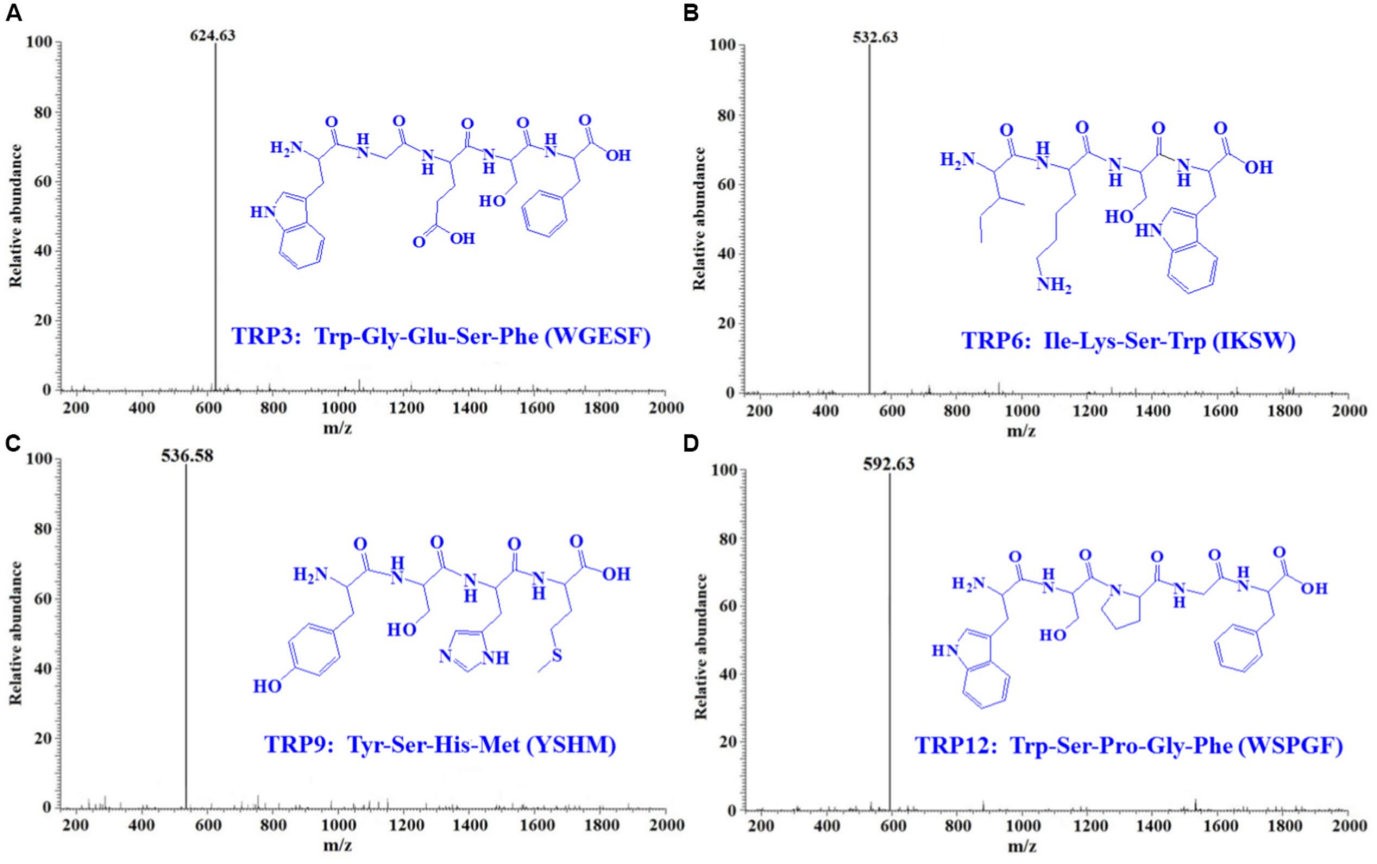
Mass spectrograms of TRP3 **(A)**, TRP6 **(B)**, TRP9 **(C)**, and TRP12 **(D)** from Skipjack tuna roe hydrolysate (STRH).

By employing Protein/Peptide Sequencer, peptide sequences of TRP3, TRP6, TRP9, and TRP12 were identified as Trp-Gly-Glu-Ser-Phe (WGESF), Ile-Lys-Ser-Trp (IKSW), Tyr-Ser-His-Met (YSHM), and Trp-Ser-Pro-Gly-Phe (WSPGF), respectively. The MWs of TRP3, TRP6, TRP9, and TRP12 were determined as 624.63, 532.63, 536.58, and 592.63 Da, respectively (Figure 4), which were in line with their theoretical MWs of 624.64, 532.63, 536.60, and 592.64 Da, respectively (Table 1 and Figure 4).

**Table 1:** Sequences, MWs, and ACEi activity (IC_50_ value) of TRP3, TRP6, TRP9, and TRP12 from Skipjack tuna roe hydrolysate (STRH).

	Retention time (min)	Amino acid sequence	Observed MW/Theoretical MW (Da)	ACEi activity (IC_50_, mg/mL)
TRP3	13.53	Trp-Gly-Glu-Ser-Phe (WGESF)	624.63/624.64	0.93 ± 0.07^a^
TRP6	15.96	Ile-Lys-Ser-Trp (IKSW)	532.63/532.63	0.79 ± 0.06^a,b^
TRP9	18.29	Tyr-Ser-His-Met (YSHM)	536.58/536.60	0.49 ± 0.06^c^
TRP12	23.65	Trp-Ser-Pro-Gly-Phe (WSPGF)	592.63/592.64	0.67 ± 0.05^d^

^a–d^Values with same letters of ACEi activity indicated no significant difference (*p* > 0.05).

### 3.4. IC_50_ values and molecular docking analysis of TRP3, TRP6, TRP9, and TRP12

Table 1 revealed that the IC_50_ value of TRP9 on ACE was 0.49 ± 0.06 mg/mL, which was significantly smaller than those of TRP3 (0.93 ± 0.07 mg/mL), TRP6 (0.79 ± 0.06 mg/mL), and TRP12 (0.67 ± 0.05 mg/mL) (*p* < 0.05). For illustrating the ACEi mechanisms of TRP3, TRP6, TRP9, and TRP12, the molecular docking experiment was performed and the affinity of TRP3, TRP6, TRP9, and TRP12 with ACE was −8.590, −9.325, −9.703, and −8.036 kcal/mol, respectively.

**Figure 5:**
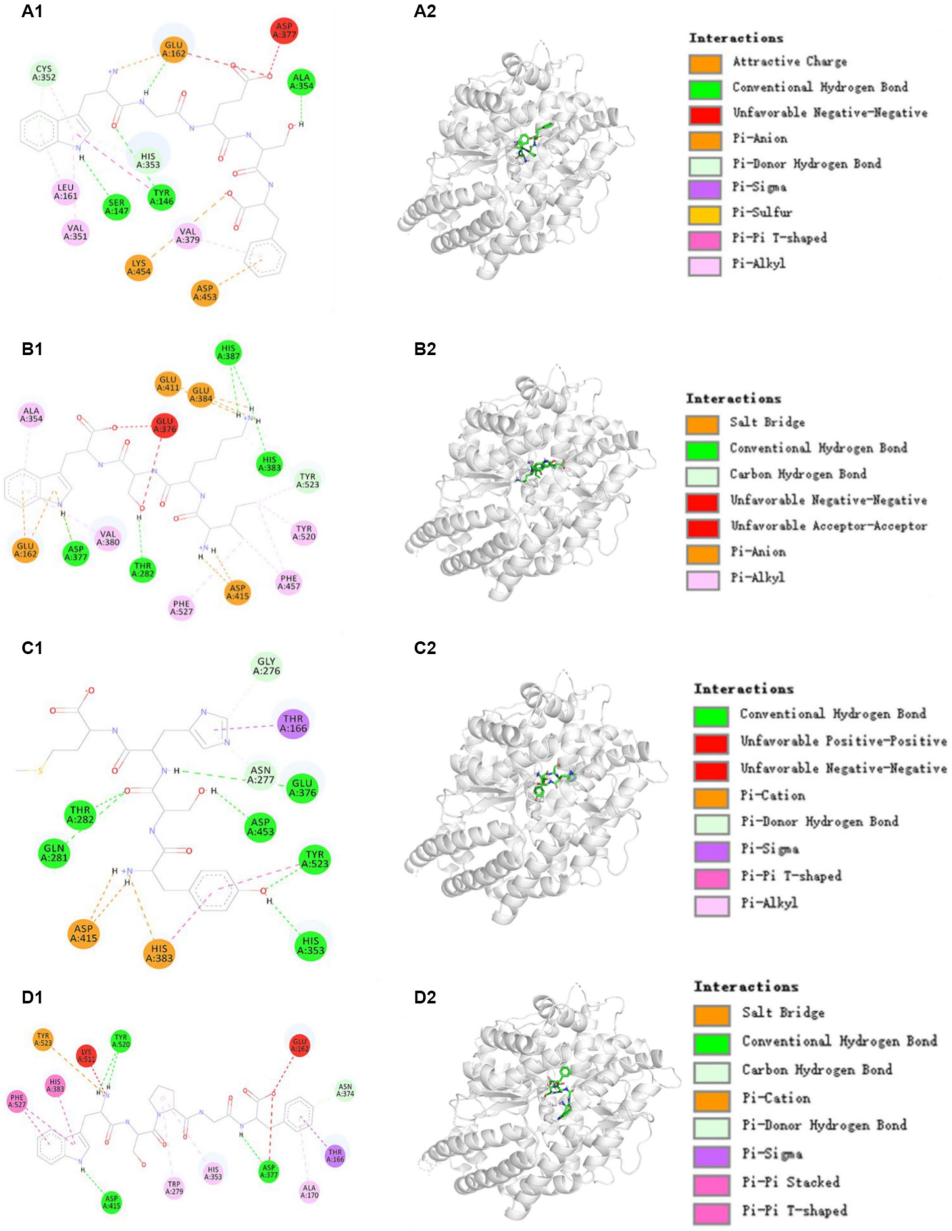
Molecular docking results of TRP3, TRP6, TRP9, and TRP12 with ACE. **(A1,A2)** 2-D and 3-D details of ACE and TRP3 interaction, **(B1,B2)** 2-D and 3-D details of ACE and TRP6 interaction, **(C1,C2)** 2-D and 3-D details of ACE and TRP9 interaction, and **(D1,D2)** 2-D and 3-D details of ACE and TRP12 interaction.

ACE has three major active site pockets (S1, S2, and S1’). S1 pocket includes Ala354, Glu384, and Tyr523 residues; S2 pocket includes Gln281, His353, Lys511, His513, and Tyr520 residues; and S1’ contains Glu162 residues (37). Cap is a widely recognized ACE inhibitor and interacts at the sites of Gln281, His353, Ala354, Glu384, Lys511, His513, Tyr520, and Tyr523 residues of ACE, which indicates that these amino acid residues play key roles in ACE binding (37). Figure 5A proved that TRP3 (WGESF) could combine with Ala354 (S1), Tyr146, Ser147, and Cys352 residues of ACE via hydrogen bonds, have hydrophobic effect with Leu161, Val351, and Val379 residues of ACE, and associate with Lys454 and Asp453 residues of ACE through electrostatic force. Figure 5B manifested that TRP6 (IKSW) could form hydrogen bonds with His383, His387, Tyr523 (S1), Thr282, and Asp377 residues of ACE, establish interaction with Ala354 (S1), Val380, Phe527, Phe457, and Tyr520 (S2) residues of ACE via hydrophobic effect, and keep in touch with Glu411, Glu384 (S1), Glu162, and Asp415 residues of ACE through electrostatic force. Figure 5C indicated that TRP9 (YSHM) could form hydrogen bonds with Thr282, Gln281 (S2), Gly276, Asn277, Glu376, Asp453, Tyr523 (S1), and His353 (S2) residues of ACE, interact with Thr166 residue of ACE through hydrophobic effect, and contact with Asp415 and His383 residues of ACE through electrostatic force. Figure 5D demonstrated that TRP12 (WSPGF) could establish hydrogen bonds with Tyr520 (S2), Asn374, Asp415, and Asp377 residues of ACE, bind with His383, Phe527, Trp279, His353 (S2), Ala170, and Thr166 residues of ACE through hydrophobic effect, and get in touch with Tyr523 (S1) residue of ACE through electrostatic force. Therefore, TRP3, TRP6, TRP9, and TRP12 displayed highlighted ACEi ability because they could effectively interact with the ACE’s active sites/pockets by hydrophobic interaction, electrostatic force and hydrogen bonding. More importantly, the ACEi capability of TRP3, TRP6, TRP9, and TRP12 were associated with the binding with ACE’s S1 (TRP3, TRP6, and TRP9) and S2 (TRP6, TRP9, and TRP12) pockets.

### 3.5. Effects of TRP3, TRP6, TRP9, and TRP12 on HUVECs

#### 3.5.1. Effects of TRP3, TRP6, TRP9, and TRP12 on cell viability

**Figure 6:**
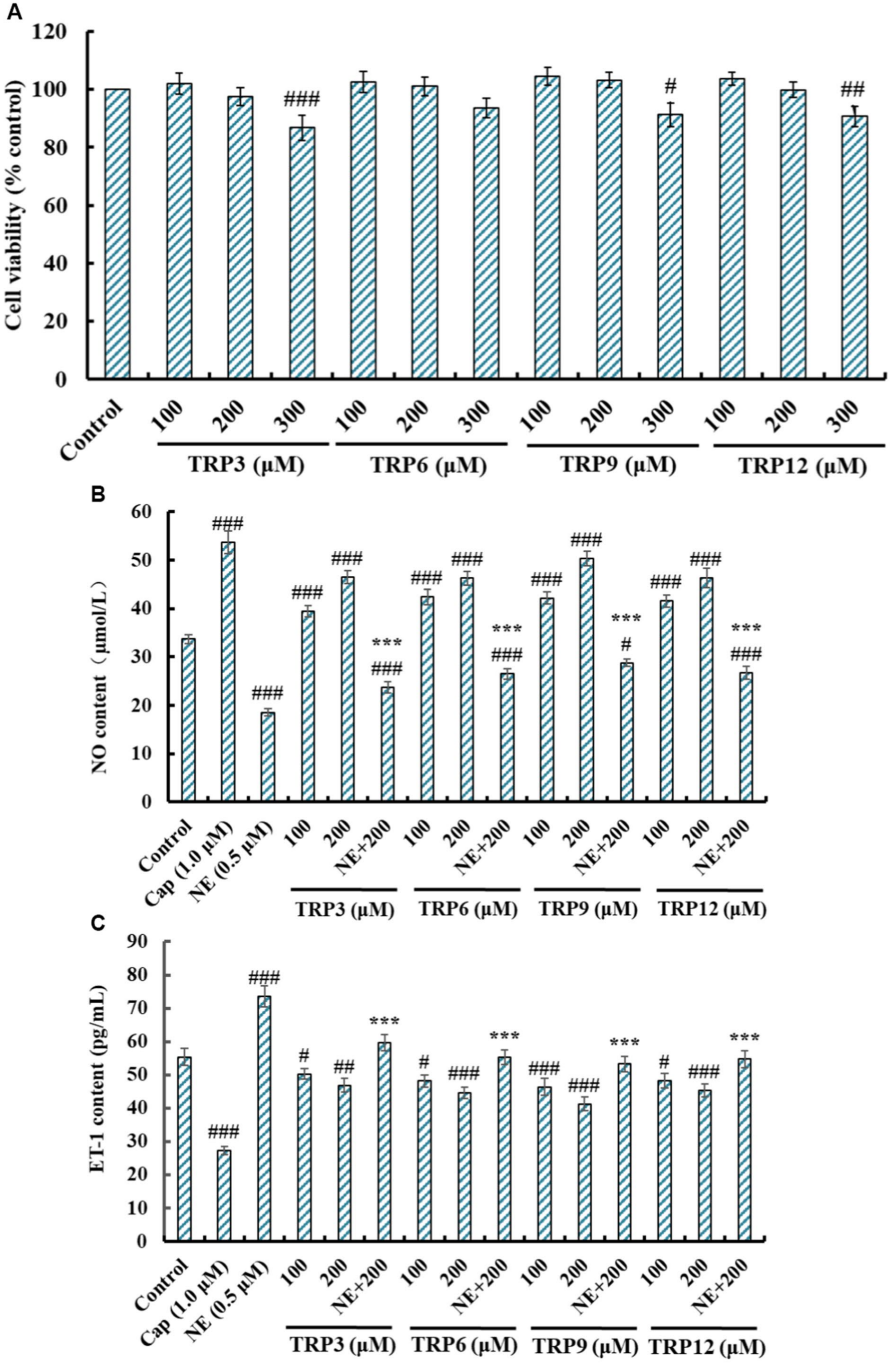
Effects of TRP3, TRP6, TRP9, and TRP12 on the cell viability, nitric oxide (NO) production (B), and endothelin-1 (ET-1) secretion (C) of HUVECs. All data are presented as the mean ± SD of triplicate results. ^###^*p* < 0.001, ^##^*p* < 0.01, and ^#^*p* < 0.05 vs. Control; ***p < 0.001 vs. Norepinephrine (NE).

The effects of TRP3, TRP6, TRP9, and TRP12 on the viability of HUVECs at 100–300 μM were shown in Figure 6A. After incubating for 24 h, the cell viability of TRP3 group ranged from 101.92 ± 3.62% to 86.72 ± 4.45%. It was important to note the cell viability of TRP3 and TRP9 groups at 300 μM was 86.72 ± 4.45% and 91.27 ± 4.05%, which was significantly smaller than those of the control and other groups. The results implied that this concentration (300 μM) might have some negative effects on the proliferation of HUVECs. Therefore, 100 and 200 μM were selected as the test concentrations of TRP3, TRP6, TRP9, and TRP12 in the follow-up experiments.

#### 3.5.2. Effects of TRP3, TRP6, TRP9, and TRP12 on No and ET-1 production

NO deficiency will give rise to the risks of CVDs, and improving the production of endothelial NO represents a good therapeutic approach for atherosclerosis (17, 24). Compared with the control group, the NO level in HUVECs was significantly increased from 33.68 ± 0.96 μM to 53.71 ± 2.313 μM by Cap treatment, but significantly decreased to 18.59 ± 0.82 μM by NE treatment (*p* < 0.001) (Figure 6B). Moreover, TRP3, TRP6, TRP9, and TRP12 could dramatically increase the NO level in HUVECs, and the NO levels treated with TRP3, TRP6, TRP9, and TRP12 at 200 μM were significantly increased to 46.52 ± 1.29, 46.25 ± 1.39, 50.34 ± 1.45, and 46.32 ± 2.03 μM. In addition, the NO content reversely lowered by NE could be, respectively, compensated to 23.69 ± 1.18, 26.51 ± 1.06, 28.79 ± 0.78, and 26.79 ± 1.34 μM, respectively, after being treated by TRP3, TRP6, TRP9, and TRP12 at 200 μM (*p* < 0.001). These data proved that TRP3, TRP6, TRP9, and TRP12 could significantly increase the NO production in HUVECs and offset in part of the decreased content by NE.

As a functional factor similar to Ang II, ET-1 can lead to endothelial dysfunction correlated with coronary heart disease and hypertension (18, 22). As depicted in Figure 6C, the ET-1 content in HUVECs was significantly decreased from 55.37 ± 2.53 pg./mL (control group) to 27.32 ± 1.28 pg./mL by Cap (1.0 μM) treatment and increased to 73.57 ± 3.09 pg./mL by NE (0.5 μM) treatment (*p* < 0.001). Furthermore, the ET-1 content in HUVECs significantly (*p* < 0.001) decreased by TRP3, TRP6, TRP9 and TRP12 at 100 and 200 μM, and the ET-1 content in TRP3, TRP6, TRP9, and TRP12 groups reduced to 46.87 ± 2.03, 44.68 ± 1.69, 41.26 ± 2.09, and 45.28 ± 1.88 pg./mL at 200 μM. In addition, the ET-1 content increased by NE was partially compensated by TRP3, TRP6, TRP9, and TRP12 treatment and decreased to 59.74 ± 2.42, 55.27 ± 2.19, 53.27 ± 2.34, and 54.71 ± 2.57 pg./mL at 200 μM (*p* < 0.001). These results illustrated that TRP3, TRP6, TRP9, and TRP12 displayed high capabilities to weaken the ET-1 production and reversed the induction of ET-1 caused by NE in HUVECs.

### 3.6. Cytoprotective functions of TRP3, TRP6, TRP9, and TRP12 on H_2_O_2_-induced HUVECs

#### 3.6.1. Influences of TRP3, TRP6, TRP9, and TRP12 on viability of H_2_O_2_-induced HUVECs

**Figure 7:**
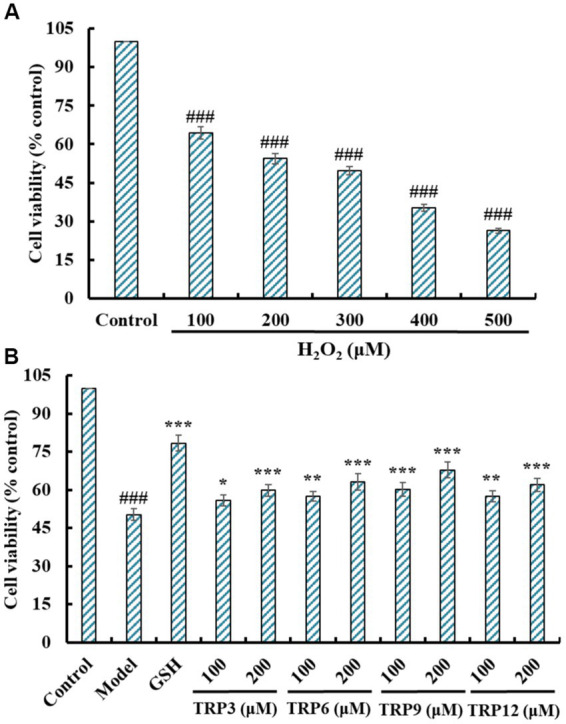
Effects on cell viability of H_2_O_2_ (100 to 500 μM) treated HUVECs **(A)** and ACEi peptides (TRP3, TRP6, TRP9, and TRP12) treated H_2_O_2_-induced HUVECs **(B)**. All data are presented as the mean ± SD of triplicate results. ^###^*p* < 0.001 vs. Control; ***p < 0.001, **p < 0.01, and *p < 0.05 vs. Model.

Literatures indicate that H_2_O_2_ concentration caused cell viability reduced to 50% is suitable for the establishment of cell oxidative damage model (13, 17). Figure 7A indicated that H_2_O_2_ could concentration-dependently reduce the cell viability of HUVECs at 100–500 μM (*p* < 0.05), and the viability of HUVECs decreased to 49.65 ± 1.68% at H_2_O_2_ concentration of 300 μM. Therefore, the concentration (300 μM) was chosen to establish the oxidative damage model of HUVECs for evaluating cytoprotective functions of TRP3, TRP6, TRP9, and TRP12.

Figure 7B presented that TRP3, TRP6, TRP9, and TRP12 could dose-dependently protected HUVECs against H_2_O_2_ damage, and the cell viability of TRP3, TRP6, TRP9, and TRP12 groups at 200 μM were increased to 59.87 ± 2.32%, 63.25 ± 3.24%, 67.85 ± 3.05%, and 61.97 ± 2.66%, respectively, which were significantly higher than that of the model group (50.31 ± 2.19%) (*p* < 0.001). However, the protective ability of TRP3, TRP6, TRP9, and TRP12 was lower than that of GSH (78.33 ± 3.08%). Then, TRP3, TRP6, TRP9, and TRP12 could dramatically increase the cell viability and give a strong protection to H_2_O_2_-induced HUVECs.

#### 3.6.2. Influences of TRP3, TRP6, TRP9, and TRP12 on ROS level in H_2_O_2_-induced HUVECs

**Figure 8:**
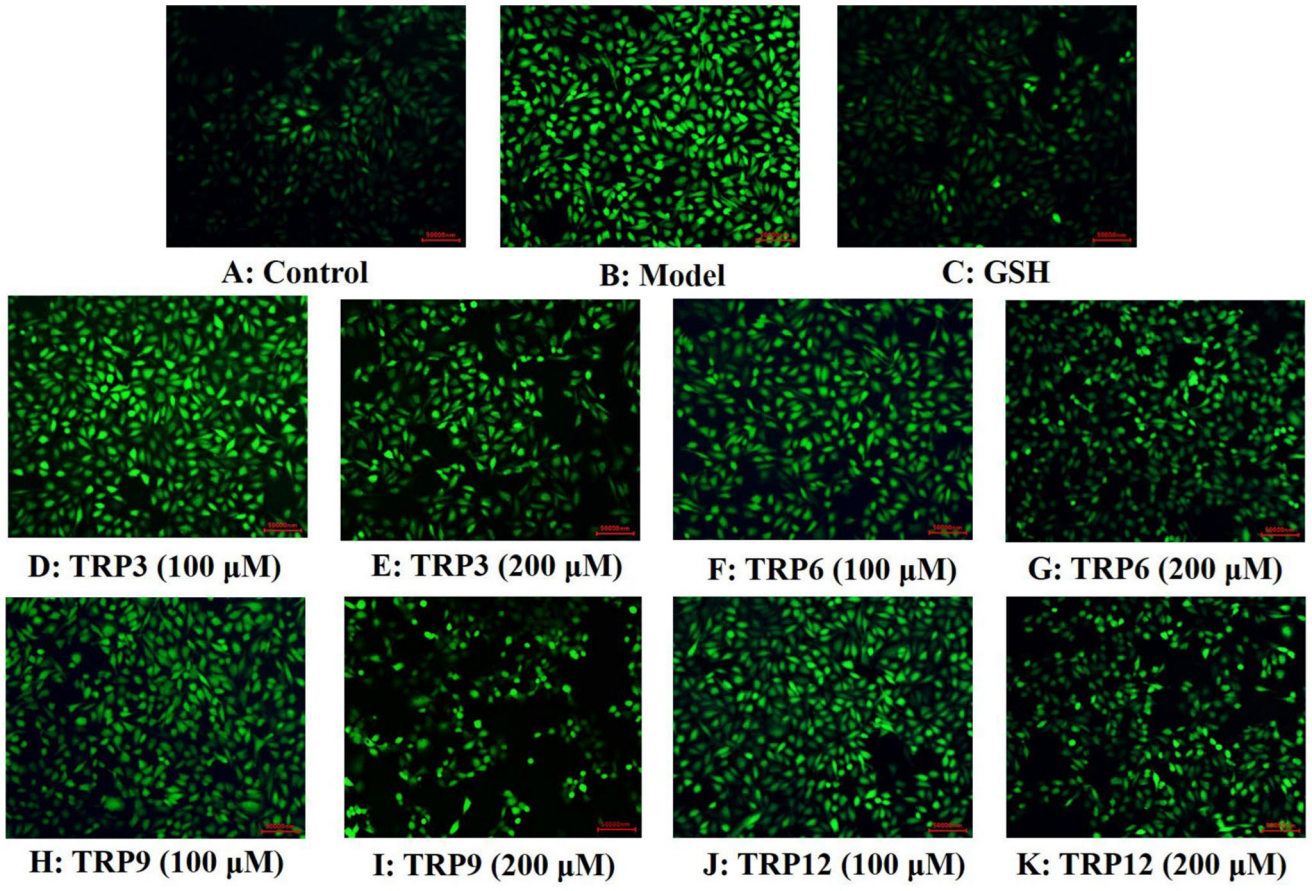
Determination of ROS contents in different groups of H_2_O_2_-induced HUVECs by DCFH-DA staining. **(A)** Control, **(B)** H_2_O_2_-induced model of HUVECs, **(C)** GSH (200 μM), **(D)–(E)** TRP3 with 100 and 200 μM, **(F)–(G)** TRP6 with 100 and 200 μM, **(H)–(I)** TRP9 with 100 and 200 μM, and **(J)–(K)** TRP12 with 100 and 200 μM.

**Figure 9:**
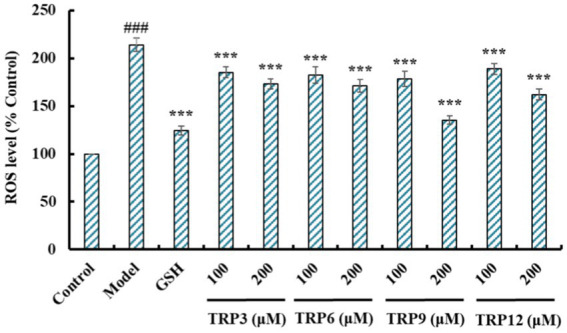
Effects of TRP3, TRP6, TRP9, and TRP12 (100 and 200 μM) on ROS levels in H_2_O_2_-induced HUVECs. All data are presented as the mean ± SD of triplicate results. ^###^*p* < 0.001 vs. Control; ***p < 0.001 vs. Model.

During oxidative stress, excessive ROS damage various cellular components and further induce apoptosis due to DNA damage, mitochondrial membrane potential reduction and enzyme inactivation (2, 50). In the model group, increased fluorescence intensity and area after DCFH-DA staining indicated a remarkable increase in ROS content of H_2_O_2_-induced HUVECs (Figure 8). Moreover, fluorescence area and intensity decreased with the increase of ACEi peptide concentration, demonstrating that TRP3, TRP6, TRP9, and TRP12 had significant ability to decrease the ROS content in H_2_O_2_-induced HUVECs. Figure 9 quantitatively determined the ability of TRP3, TRP6, TRP9, and TRP12 to decrease the ROS content in the H_2_O_2_-induced HUVECs. At 200 μM, the ROS levels of TRP3, TRP6, TRP9, and TRP12 groups were significantly decreased from 146.6 ± 3.81% to 128.7 ± 5.2%, 127.5 ± 4.32%, 122.4 ± 4.47%, and 123.7 ± 3.21% control, respectively. Therefore, ROS levels in H_2_O_2_-induced HUVECs were significantly decreased by pretreatment of TRP3, TRP6, TRP9, and TRP12.

#### 3.6.3. Influences of TRP3, TRP6, TRP9, and TRP12 on antioxidase activity, NO production, and MDA content in H_2_O_2_-induced HUVECs

**Figure 10:**
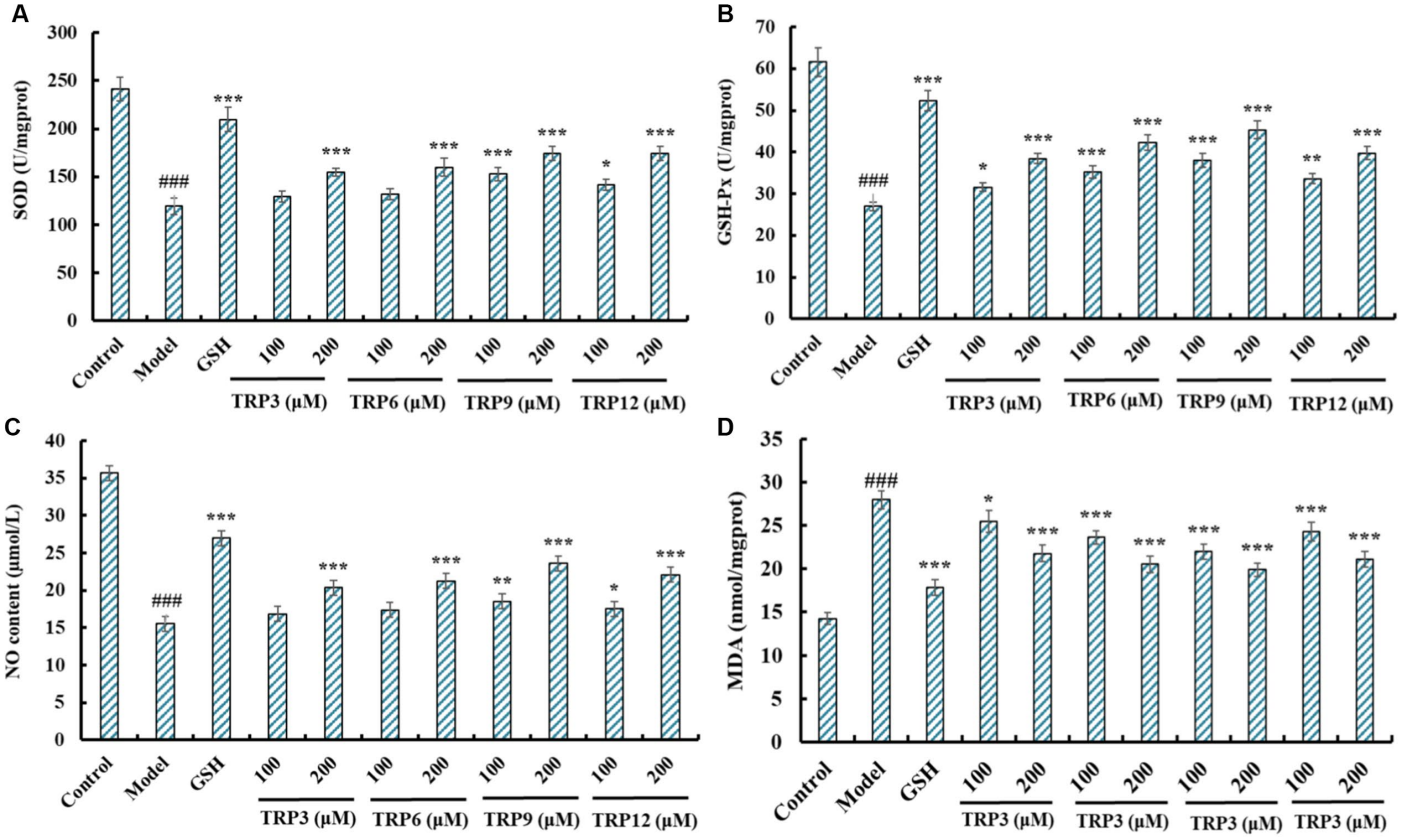
Effects of TRP3, TRP6, TRP9, and TRP12 on the levels of SOD **(A)**, GSH-Px **(B)**, NO **(C)**, and MDA **(D)** of H_2_O_2_-induced HUVECs. All data are presented as the mean ± SD of triplicate results. ^###^*p* < 0.001 vs. Control; ***p < 0.001, **p < 0.01, and *p < 0.05 vs. Model.

Excess ROS were produced in H_2_O_2_-induced HUVECs, and the activity of antioxidases is the key to the scavenging efficiency of ROS (40, 50). At 200 μM, SOD activity in TRP3, TRP6, TRP9, and TRP12 groups was 154.58 ± 3.94, 160.02 ± 9.04, 174.32 ± 7.26, and 174.32 ± 7.42 U/mg prot, respectively, which was significantly higher than that of the model group (108.57 ± 7.56 U/mg prot) (*p* < 0.001) (Figure 10A). The influences of TRP3, TRP6, TRP9, and TRP12 on GSH-Px activity were similar to that of SOD (Figure 10B). At 200 μM, the GSH-Px activity in TRP3, TRP6, TRP9, and TRP12 groups was 38.46 ± 1.25, 42.35 ± 1.86, 45.36 ± 2.07, and 39.75 ± 1.63 U/mg prot, respectively, which was observably greater than that of the model group (26.95 ± 1.09 U/mg prot) (*p* < 0.001).

Figure 10C revealed that the NO production was significantly elevated after pretreating with TRP3, TRP6, TRP9, and TRP12 compared with model group (15.54 ± 0.68 μM) (*p* < 0.001). At 200 μM, the NO contents in TRP3, TRP6, TRP9, and TRP12 groups were 20.38 ± 0.67, 21.26 ± 0.59, 23.64 ± 0.82, and 22.09 ± 0.81 μM, respectively. However, the ability of TRP3, TRP6, TRP9, and TRP12 to promote NO production was inferior to that of GSH (26.98 ± 1.05 μM). Figure 10D depicted that TRP3, TRP6, TRP9, and TRP12 could significantly lower the MDA content of H_2_O_2_-induced HUVECs compared with model group (27.94 ± 1.06 nmol/mg prot) (*p* < 0.001). At 200 μM, the MDA contents in TRP3, TRP6, TRP9, and TRP12 groups were 21.76 ± 0.94, 20.52 ± 0.92, 19.88 ± 0.75, and 21.08 ± 0.92 nmol/mg prot, respectively. However, the ability of TRP3, TRP6, TRP9, and TRP12 to reduce MDA production was inferior to that of GSH (17.83 ± 0.87 nmol/mg prot). Therefore, TRP3, TRP6, TRP9, and TRP12 can increase the production of NO and activity of GSH-Px and SOD to reduce the production of MDA.

#### 3.6.4. Effects of TRP3, TRP6, TRP9, and TRP12 on the apoptosis rates of H_2_O_2_-induced HUVECs

**Figure 11:**
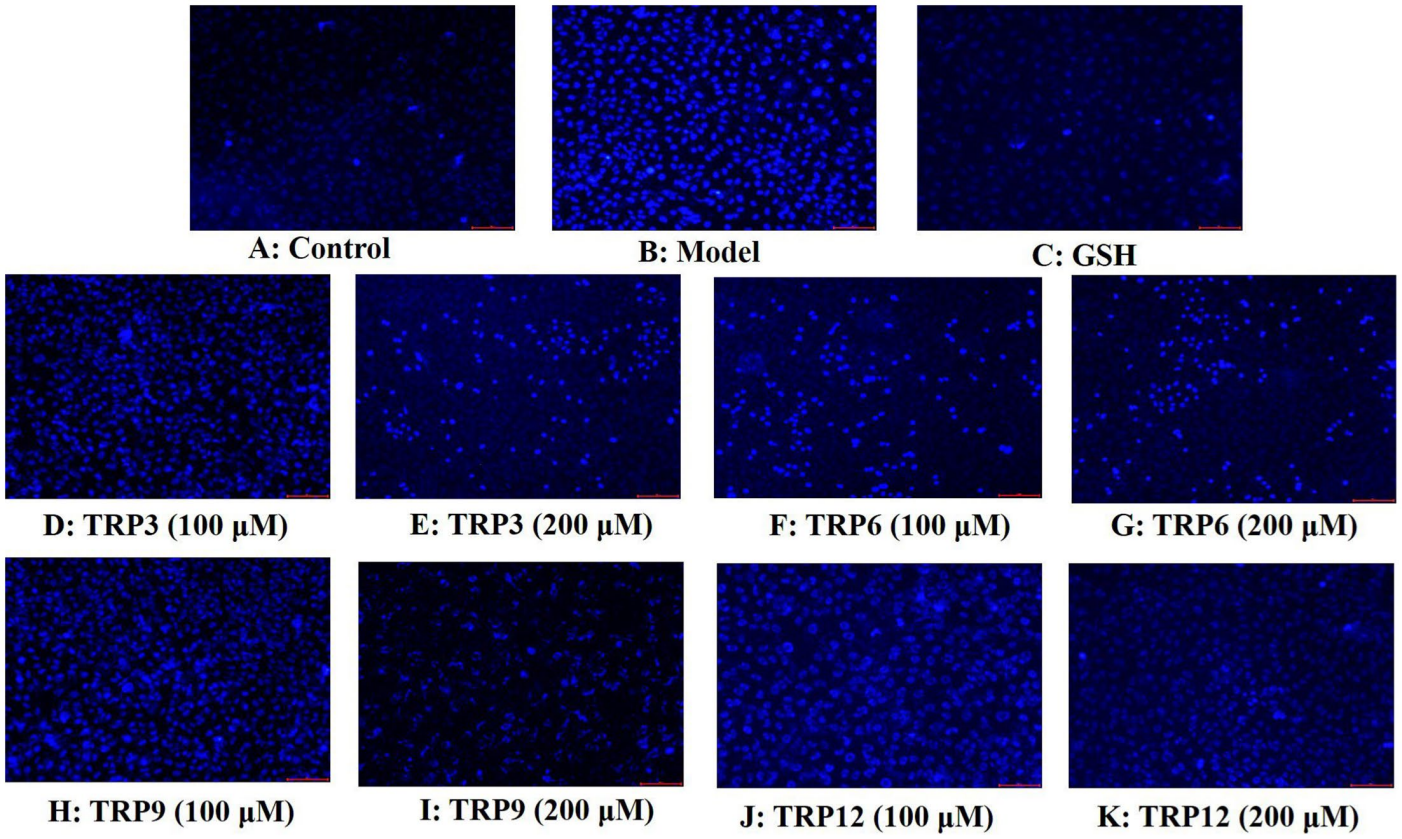
Apoptosis analysis of TRP3, TRP6, TRP9, and TRP12 on H_2_O_2_-induced HUVECs by Hoechst 33342. GSH (200 μM) served as the positive control. **(A)** Control, **(B)** H_2_O_2_-induced HUVECs, **(C)** GSH, **(D)–(E)** TRP3 with 100 and 200 μM, **(F)–(G)** TRP6 with 100 and 200 μM, **(H)–(I)** TRP9 with 100 and 200 μM, and **(J)–(K)** TRP12 with 100 and 200 μM.

**Figure 12:**
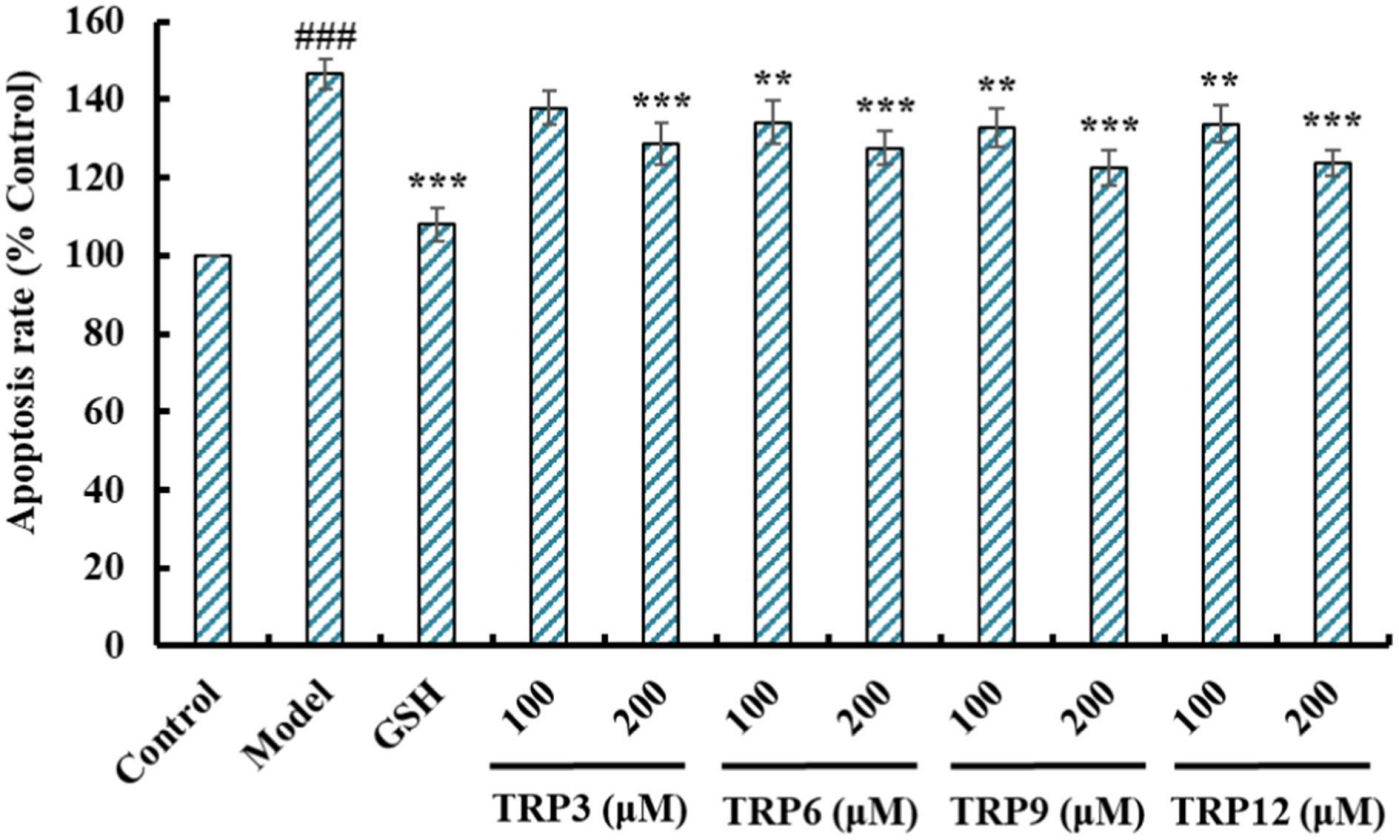
Effects of TRP3, TRP6, TRP9, and TRP12 on apoptosis rates of H_2_O_2_-induced HUVECs. GSH (200 μM) served as the positive control. All data are presented as the mean ± SD of triplicate results. ^###^*p* < 0.001 vs. Control; ***p < 0.001, **p < 0.01, and *p < 0.05 vs. Model.

Compared with the control group (Figure 11A), HUVECs in the model group (Figure 11B) displayed a larger area of blue fluorescence and were in a dense dye state, which proved that H_2_O_2_ caused a great deal of HUVECs in an apoptosis state. Nevertheless, the fluorescence area and intensity of the TRP3, TRP6, TRP9, and TRP12 groups gradually decreased at 100 and 200 μM (Figures 11D–K). In addition, TRP9 showed a stronger inhibiting ability on H_2_O_2_-induced apoptosis of HUVECs than TRP3, TRP6, and TRP12. Figure 12 displayed that the apoptosis rates of H_2_O_2_-damaged HUVECs in TRP3, TRP6, TRP9, and TRP12 groups were significantly brought down compared with the model group (214.03 ± 7.11% control) (*p* < 0.01). At 200 μM, the total apoptosis ratios in TRP3, TRP6, TRP9, and TRP12 groups were 173.12 ± 5.11%, 171.21 ± 6.92%, 135.37 ± 4.28%, and 162.08 ± 5.94% control, respectively. However, the ability of TRP3, TRP6, TRP9, and TRP12 on reducing apoptosis of H_2_O_2_-damaged HUVECs was inferior to that of GSH (124.52 ± 4.82% control). The current results manifested that TRP3, TRP6, TRP9, and TRP12 could significantly protect HUVECs against H_2_O_2_ damage by reducing apoptosis.

## 4. Discussion

### 4.1. Production of ACEi peptides from Skipjack tuna roes

BPs released from food proteins may have high ACEi ability and alleviate cellular oxidative damage, which are two key ways to treating hypertension (17, 21). BPs hide in parent proteins and can be released by chemical degradation, proteinase hydrolysis, and microbiological fermentation methods (20, 33). Proteinase hydrolysis is a popular process because of its multiple advantages, such as easily controlling the process, no pollution to the environment, and no toxic chemical residues (51, 52). Because of the specificity of proteases, proteins can produce multiple hydrolysates with diverse bioactivities. Therefore, endonuclease, exonuclease, and their combinations serve as tools to yield BPs from marine creatures and their byproducts (53, 54). In the experiment, we employed six proteases to hydrolyze roe proteins of Skipjack tuna, and the hydrolysate generated by flavourzyme exhibited the highest ACEi capacity (Figure 2), which further proved that the specificity of proteases can greatly affect the kind of peptides in hydrolysates, which were closely correlated with their physiological and pharmacological functions.

Protein hydrolysates are made up of diverse peptides with different MWs and physicochemical properties because of the differences in amino acid composition, which are major factors determining the methods of peptide separation (55). Large polypeptides difficultly access the molecular pockets of ACE and combine with its active sites, leading to a decrease of inhibitory ability (21, 37). For this reason, ultrafiltration and gel permeation chromatography are popularly applied to collect and isolate BPs with short chains from protein hydrolysates, such as *Mustelus* (14), *Cyclina sinensis* (56), Antarctic krill (18), tuna frame (57) and milts (44), *Ulva prolifera* (58), and *Arthrospira platensis* (59). In addition, BPs have differences in ion exchange and polarity ability due to the polar groups of amino acids, such as amino and carboxyl groups. Thereby, ion exchange chromatography and RP-HPLC are also known as common techniques for peptide purification (53, 60). According to these literatures, we designed the separation process of tuna roe hydrolysate and four ACEi peptides, including TRP3 (WGESF), TRP6 (IKSW), TRP9 (YSHM), and TRP12 (WSPGF), were prepared and showed significant ACEi ability.

### 4.2. Structure–activity relationship of TRP3, TRP6, TRP9, and TRP12

The IC_50_ values of TRP3, TRP6, TRP9, and TRP12 were 0.93 ± 0.07, 0.79 ± 0.06, 0.49 ± 0.06, and 0.67 ± 0.05 mg/mL, respectively, which were lower than those of ACEi peptides, such as LAPPSTRH (1.31 mg/mL) (61), GPWGPA (24), VAP (1.71 mg/mL) (62), MVGSAPGVL (3.09 mg/mL) (63), FRKE (6.97 mg/mL) (18), FQK (1.73 mg/mL) (22), YLRLHF (1.26 mg/mL) (64), NFRPQ (4.28 mg/mL) (17), and FEM (2.18 mg/mL) (37), etc.

Peptide size and amino acid sequence are two key factors affecting the ACEi ability of BPs (17, 21). Peptide size decides the affinity between BPs and ACE because big polypeptides cannot be accommodated in and access the narrow channel of ACE (21). For example, VPP and IPP can easily access and bind the Zn^2+^ of ACE channel, but ALPMHIR has low binding scores with ACE because it uncomfortably accesses the narrow channel (19). In the experiment, TRP3, TRP6, TRP9, and TRP10 are tetrapeptides or pentapeptides, and small size increases their access to the active channel of ACE, and this has been proved that the affinities of TRP3, TRP6, TRP9, and TRP12 with ACE were −8.590, −9.325, −9.703, and −8.036 kcal/mol, respectively, which were similar to those of ACEi peptides, such as SHGEY (−8.7 kcal/mol), SPYGF (−9.7 kcal/mol) (24), LSFR (−8.5 kcal/mol), IYSP (−8.3 kcal/mol) (17), YSK (−7.9 kcal/mol) (65), YEGDP (−8.8 kcal/mol), SWISS (−8.6 kcal/mol) (44), YVVF (−9.8 kcal/mol), WMY (−9.3 kcal/mol), and LVLL (−8.6 kcal/mol) (66).

Amino acids are another factor affecting the ability of ACEi peptides. Chen et al. (64) reported that hydrophobic amino acids (Met, Ile, Phe, Trp or Lys) could significantly contribute to the inhibitory potency of peptide fraction from bighead carp (*Aristichthys nobilis*) hydrolysates. Moreover, the C- and N-terminal amino acids are believed to play a crucial function in the activity of ACEi peptides (21). Yu et al. (56) reported that C-terminus (such as Trp, Tyr, Pro, or Phe) and N-terminus (such as Pro, Phe, Trp, or Met) hydrophobic amino acid residues have positive effects on the ACEi activity of BPs, and the ACEi activity of WPMGF is due to the Phe and Trp residues at C- and N-terminus. Su et al. (67) found that the presence of aromatic and hydrophobic amino acids at the C- and N-terminus could significantly enhance the ACEi ability of PPLLFAAL. Wang et al. (65) concluded that YSK (IC_50_: 76 μM) and YPK (IC_50_: 38.7 μM) showed better ACEi activity than KFYG (IC_50_: 90.5 μM) and ACKEP (IC_50_: 126 μM) were due to the same N- and C-terminal amino acids (Tyr or Lys). Suo et al. (17) reported that C-terminus (Lys, Pro, or Phe) and N-terminus (Ile or Tyr) amino acids were particularly critical for the ACEi activity of IK, YEGDP and WF. Therefore, the N- and C-terminal amino acids of TRP3 (Phe and Trp), TRP6 (Ile and Trp), TRP9 (Tyr and Met), and TRP12 (Trp and Phe) are particularly important for their ACEi activity.

### 4.3. Cytoprotection of TRP3, TRP6, TRP9, and TRP12 on HUVECs

ECs are a constitutive part of the heart and vasculature and generally believed that ECs activation and dysfunction are preliminary processes in the pathological processes of CVDs including high blood pressure, AS and heart failure (16). Then, HUVECs commonly serve as model cells for illustrating the mechanism and developing new drugs for CVDs. In addition, EC proliferation is vital for forming new vessels, and ECs also serve as the therapeutic target of CVDs (68). Figure 8 indicated that TRP3, TRP6, TRP9, and TRP12 had no significant toxicity to HUVEC at 100 and 200 μM, which affirmed that they were appropriate for application in health products treating CVDs at concentrations below 200 μM.

NO refers to the most potent vascular endothelium factor and the deficiency of NO will raise the risks of CVDs and AS in pathologic situations. Improvement of endothelial NO production represents effectively curing strategies for AS (17). Therefore, some ACEi peptides, such as YEGDP (17), GRVSNCAA (27), LPRS (22), KYIPIQ (69), DIGGL (58), SHGEY (24), and IVTNWDDMEK (70), exerted their antihypertensive functions via increasing the NO production of HUVECs. ET-1 is a well-known vasoconstrictor analogous to Ang II and can cause endothelial dysfunction correlated with hypertension and AS (18). ACEi peptides, including TYLPVH (27), MKKS (22), VDRYF (37), SPYGF (24), DIGGL (58), and VGPAGPRG (70), exerted their anti-hypertensive functions via memorably weakening ET-1 generation. The present results demonstrated that TRP3, TRP6, TRP9, and TRP12 could effectively protect ECs, and the mechanism was concerned with improving the level of NO, weakening the generation of ET-1, and combating the negative impact of NE on NO and ET-1 production in HUVECs.

Oxidative stress represents the primary inducement of endothelial dysfunction, and it further leads to injuring the barrier function of vascular endothelium and the pathogeny of AS, hypertension and other CVDs. Additionally, high ROS content badly harms a number of functioning cell components, lowers membrane potential, deactivates antioxidant enzymes, and even results in transgene, which causes HUVECs to undergo apoptosis (47). Therefore, oxidative stress is the primary determinant for EC activation and dysfunction, and apoptosis is another main mechanism of EC injury caused by oxidative stress (17). Therefore, we established the oxidative damage model of HUVECs using 300 μM H_2_O_2_ for exploring the protective capacity and mechanisms of TRP3, TRP6, TRP9, and TRP12 on EC oxidative injury.

To keep the cells in tip-top condition, the antioxidative defense system will get started timely to remove excessive ROS (70, 71). MDA is a key peroxidation metabolite of the cell membrane lipid and acts as a proverbial referent to evaluate the degree of oxidative damage (55, 72). ACEi peptides of IVTNWDDMEK and VGPAGPRG from *Volutharpa ampullacea* perryi can dose-dependently regulate NO and ET-1 generation and protect HUVECs against H_2_O_2_-induced injury, and mechanisms indicate that IVTNWDDMEK and VGPAGPRG can up-regulate the expression of Nrf2 and HO-1 to reduce the accumulation of ROS and MDA (70). FNLRMQ from *Takifugu bimaculatus* can be used as a potential candidate compound for alleviating the viability and apoptosis of Ang-II-induced HUVECs by regulating Nrf2/HO-1 and PI3K/Akt/eNOS signaling pathways (73). FEIHCC and EMFGTSSET from *Isochrysis zhanjiangensis* can alleviate endothelial damage by blocking inflammation and apoptosis of HUVECs, and mechanisms demonstrate that EMFGTSSET can regulate MAPK/NF-κB/Akt signal pathways to reduce the ROS and related cytokines (74, 75). The current results turned out that the protective functions of TRP3, TRP6, TRP9, and TRP12 to H_2_O_2_-damaged HUVECs were parallel to those BPs, and their mechanisms were connected with activating Nrf2 pathway to reduce the oxidative stress level and apoptosis rate of H_2_O_2_-induced HUVECs.

## 5. Conclusion

In summary, fifteen peptides were purified from the roe hydrolysate of Skipjack tuna generated by employing flavourzyme, and four peptides with remarkable ACEi ability were identified as WGESF, IKSW, YSHM, and WSPGF, respectively. WGESF, IKSW, YSHM, and WSPGF displayed remarkable hypotensive effects via inhibiting ACE activity and regulating NO and ET-1 production in HUVECs. In addition, WGESF, IKSW, YSHM, and WSPGF could lower the oxidative stress damage and apoptosis rate of H_2_O_2_-damaged HUVECs by increasing the levels of SOD, GSH-Px, and NO to decrease the production of ROS and MDA. Therefore, this study is not only to develop a technology for the production of novel ACEi peptides of skipjack tuna roes, but also to be conducive to dealing with the problem of environmental pollution induced by tuna byproducts. Another even more important is that WGESF, IKSW, YSHM, and WSPGF might be used as natural functional ingredients for developing noticeable hypotensive products to ameliorate hypertension and CVDs. In addition, the mechanisms of WGESF, IKSW, YSHM, and WSP GF for ameliorating hypertension and cardiovascular diseases will be further investigated by *in vivo* experiments.

## Data availability statement

The raw data supporting the conclusions of this article will be made available by the authors, without undue reservation.

## Author contributions

W-YZ, Y-MW, M-XG, H-WW, and S-LZ: data curation, methodology, and formal analysis. H-YZ: conceptualization, methodology, and funding acquisition. BW: supervision, funding acquisition, and writing-review and editing. All authors contributed to the article and approved the submitted version.

## Funding

This work was funded by the National Natural Science Foundation of China (No. 82073764) and Ten-thousand Talents Plan of Zhejiang Province (No. 2019R52026).

## Conflict of interest

H-WW was employed by Ningbo Today Food Co., Ltd.

The remaining authors declare that the research was conducted in the absence of any commercial or financial relationships that could be construed as a potential conflict of interest.

## Publisher’s note

All claims expressed in this article are solely those of the authors and do not necessarily represent those of their affiliated organizations, or those of the publisher, the editors and the reviewers. Any product that may be evaluated in this article, or claim that may be made by its manufacturer, is not guaranteed or endorsed by the publisher.

## Figures and Tables

**Table:** 

ACE	Angiotensin-I-converting enzyme inhibitory
HUVECs	Human umbilical vein endothelial cells
NO	Nitric Oxide
ET-1	Endothelin-1
SOD	Superoxide dismutase
GSH-Px	Glutathione peroxidase
ROS	Reactive oxygen species
MDA	Malondialdehyde
BPs	Bioactive peptides
CVDs	Cardiovascular diseases
AS	Atherosclerosis
Ang	Angiotensin
Cap	Captopril
ECs	Endothelial cells
GSH	Glutathione
FBS	Fetal bovine serum
NE	Norepinephrine
TFA	Trifluoro acetic acid
